# Traditional Chinese Medicine Shenmayizhi Decoction Ameliorates Memory and Cognitive Impairment Induced by Multiple Cerebral Infarctions

**DOI:** 10.1155/2021/6648455

**Published:** 2021-03-30

**Authors:** Chengcheng Sun, Jiangang Liu, Nannan Li, Meixia Liu, Zenggang Luo, Hao Li

**Affiliations:** ^1^Xiyuan Hospital, China Academy of Chinese Medical Sciences, Beijing 100091, China; ^2^Beijing Administration of Traditional Chinese Medicine, Beijing 100053, China

## Abstract

This study aimed to illustrate the mechanism by which Shenmayizhi decoction (SMYZD) improves the learning memory of rats with vascular cognitive impairment (VCI). Fifty male and female Wistar rats of specific pathogen-free grade (SPF grade) were used to establish the model by the administration of a microsphere embolization. This was accomplished by injecting sterile, standardized, mass-produced microspheres of uniform particle size (100–200 *µ*m in diameter) in a sodium alginate microsphere vascular embolic agent suspension to induce VCI. The VCI model was successfully established in 40 rats, including both male and female rats, and the rats were randomly divided into 4 groups of 10 rats each. The model group was administered an equal volume of distilled water. The donepezil group was administered 0.45 mg/kg/d donepezil, which is equivalent to the clinical dosage. The SMYZ-H group was administered 11.88 g/kg/d SMYZ, which is 4 times higher than the clinically equivalent dosage. The SMYZ-L group was administered 2.97 g/kg/d SMYZ, which is the clinically equivalent dosage. A sham-operated group was used as the control group and administered an equal volume of distilled water. The rats in the 4 groups were treated by gavage with equal volumes of liquid and the indicated concentration of drug diluted in distilled water for 8 consecutive weeks. Two months later, the Morris water maze (MWM) was used to evaluate the spatial memory of all the rats. Ultrastructural and ultrapathological changes in the capillaries of the cerebral cortex were observed by transmission electron microscopy. Furthermore, Western blot and RT-PCR analyses were used to assess the levels of platelet-derived growth factor receptor-*β* (PDGFR-*β*), neuron-glial antigen 2 (NG2), vascular endothelial growth factor A (VEGF-A), and angiopoietin 1 (Ang1) in the cerebral cortex of the rats. The results showed that SMYZD at concentrations of 11.88 g/kg/d and 2.97 g/kg/d (SMYZ-H and SMYZ-L) significantly shortened the escape latency (EL). In addition, SMYZ-H significantly prolonged the distance traveled and the time spent in the original platform quadrant by the rats with VCI. SMYZ-H significantly increased the NG2 and Ang1 protein expression levels and increased the PDGFR-*β* and Ang1 mRNA levels. These results demonstrated that Shenmayizhi decoction can improve the memory abilities of rats with VCI induced by multiple cerebral infarctions by preventing pericyte degeneration.

## 1. Introduction

Vascular cognitive impairment (VCI) is a type of cognitive disorder associated with cerebrovascular disease (CBVD) [1] and encompasses the full range of cognitive deficits from mild cognitive impairment to dementia. The etiology of VaD mainly includes two parts, namely, cerebrovascular disease and risk factors. The risk factors for vascular dementia include cerebrovascular disease, stroke, white matter ischemic lesions, advanced age, and low education level [2, 3]. VCI is the second most common neuropathology associated with dementia, accounting for up to one-third of the population risk. Importantly, in people with dementia, VCI often coexists with AD [4]. Autopsy studies show that vascular brain injury accounts for 33% of the risk of dementia [5]. Vascular dementia is now recognized as the second most common form of dementia following Alzheimer's disease [6], which makes VCI a substantial public health problem. However, VCI is still considered to be a type of dementia that can be prevented, and it may be reversible with early intervention. There is increasing awareness that targeting vascular risk may help prevent dementia. Exploring the pathogenesis of VCI will pave the way for the development of treatments that target the underlying disease-related processes.

The pathological mechanisms of VCI may include impaired pericyte and endothelial cell function and impaired cytokine and vascular factor production. Pericytes may play an important role in causing the memory deficits of VCI. No single event can be considered the sole cause of cognitive impairment in VCI. These events are interrelated and can have cascading effects that lead to cognitive impairment [7]. Pericytes (PCs) were first named Rouget cells and were then renamed “pericytes” due to their unique localization in the perivascular space of brain vessels [8]. The main function of pericytes is to regulate the permeability of the blood-brain barrier and the clearance and phagocytosis of cell debris [9]. Vascular factors can lead to a loss of peripheral cells, which in turn causes endothelial cell death and exacerbates vascular dysfunction, leading to the degeneration of brain microvessels and chronic hypoxia and promoting the death of neurons.

PC markers, such as platelet-derived growth factor receptor-*β* (PDGFR-*β*), neuron-glial antigen 2 (NG2), vascular endothelial growth factor A (VEGF-A), and angiopoietin 1 (Ang1), reflect the function of pericytes and play important regulatory roles in angiogenesis [10–12].

Traditional Chinese medicine (TCM) has been used for centuries for the treatment of VCI due to its multiple pharmacological effects, safety, and low cost in China. Shenmayizhi Decoction is composed of Panax ginseng C.A. Mey (Renshen), *Gastrodia elata* Bl. (Tianma), *Euonymus alatus* (Thunb.) Sieb. (Guijianyu), and rhizome of *Ligusticum chuanxiong* Hort. (Chuanxiong), and it is used as a clinical protocol for treating VCI patients at Xiyuan Hospital of China Academy of Chinese Medical Sciences. Previous clinical trials and animal experiments conducted by our research group have shown that SMYZD has a significant effect on the treatment of dementia [13–16].

In this study, we generated a VCI model of multiple cerebral infarctions induced by the injection of a sodium alginate microsphere vascular embolic agent suspension (KMG). The effect of SMYZD on spatial learning and memory was examined, and the differences in PDGFR-*β*, NG2, VEGF-A, and Ang1 expression were evaluated by PCR and Western blotting to illustrate the pathological changes of pericytes. We aimed to explore the relationship between cerebral microvascular pericyte degeneration and vascular cognitive impairment, the regulatory mechanism underlying the effects of SMYZD, and the correlation of SMYZD with cerebral microvascular pericytes.

## 2. Materials and Methods

### 2.1. Animals

Fifty male and female specific pathogen-free grade (SPF grade) Wistar rats weighing 200–220 g were obtained from the Chinese Academy of Medical Science (license number: SCXK (Beijing) 2016–0006). The experiment was carried out after 7 days of adaptive feeding in a room where the temperature was controlled at 24°C∼26°C and the humidity was controlled at 40%∼70%. The animal model and experiment processes were performed in strict accordance with the recommendations in the Guide for the Care and Use of Laboratory Animals of the National Institutes of Health. This experiment was approved by the Committee on Ethics of Animal Experiments of Xiyuan Hospital of China Academy of Chinese Medical Sciences (No. 2018XLC004-2).

### 2.2. Drug Preparation

SMYZD extract (2.21 g/g) was prepared in the preparation room of Xiyuan Hospital, China Academy of Chinese Medical Sciences. Pieces of Chinese herbs, including Panax ginseng C.A. Mey (Renshen), *Gastrodia elata* Bl. (Tianma), *Euonymus alatus* Sieb. (Thunb.) (Guijianyu), and *Ligusticum chuanxiong* Hort. (Chuanxiong), were obtained from Hebei Shennong Pharmaceutical Co., Ltd. (Beijing). Donepezil hydrochloride (Aricept, 5 mg/tablet, batch number: 1702012) was produced by Eisai Pharmaceutical Co., Ltd. (China).

### 2.3. Reagents

Sodium alginate microspheres for vascular embolism (KMG) (100–200 *µ*m, batch number: 20150809) were produced by Beijing Shengyiyao Technology & Development Co., Ltd. HRP Affini Pure goat anti-rabbit IgG (*H* + *L*) (batch number: S004) and HRP Affini Pure goat anti-mouse IgG (*H* + *L*) (batch number: S001) antibodies were produced by Beijing TDY Biotech Co., Ltd. GAPDH mouse monoclonal antibodies (batch number: YM3029) were produced by Immunoway Biotechnology Co., Ltd. TRIzol Universal RNA Extraction Reagent (batch number: DP405-02) was produced by Tiangen Biotech Co., Ltd. (Beijing). Prime Script RT reagent Kit with gDNA Eraser (batch number: RR82LR), SYBR Premix Ex Taq II (Tli RNaseH Plus), ROX plus (batch number: RR82LR), and DL2000 DNA Marker were produced by Takara Biotechnology Co., Ltd. PDGR-DGR dhn monoclonal antibody (batch number: GR212663-60) and NG2 polyclonal antibody (batch number: GR3205582-1) were produced by Abcam Co., Ltd.

### 2.4. SMYZD Extract Preparation

Slices of the herb SMYZD (composition: *Panax ginseng* C.A. Mey (Renshen), *Gastrodia elata* Bl. (Tianma), *Euonymus alatus* (Thunb.) Sieb. (Guijianyu), and rhizome of *Ligusticum chuanxiong* Hort. (Chuanxiong) at a ratio of 3 : 3 : 3 : 2) were prepared in the preparation room of Xiyuan Hospital of China Academy of Chinese Medical Sciences, Beijing Hospital: Z20200005000. Ginseng slices were decocted alone and with water twice: first with 12 times the amount of water for 2 hours and then with 10 times the amount of water for 2 hours. The decoctions were filtered and combined. *Gastrodia elata* Bl. (Tianma), *Euonymus alatus* (Thunb.) Sieb. (Guijianyu), and rhizome of *Ligusticum chuanxiong* Hort. (Chuanxiong) were decocted with water three times: 2 times using 10 times the amount of water for 2 hours and 1 time using 8 times the amount of water for 1 hour. The filtrate was combined with the ginseng filtrate, and the filtrate was concentrated to produce an extract with a relative density of 1.10 ± 1.15 (50°C) [17]. Each gram of SMYZD extract contained 2.21 g of crude drug.

### 2.5. Animals Groups and Drug Administration

Forty rats with VCI, half male and half female, were randomly divided into 4 groups with 10 rats per group. The model group was administered an equal volume of distilled water. The donepezil group was administered 0.45 mg/kg/d donepezil, which is equivalent to the clinical dosage. The dosages of SMYZD were calculated on the basis of the body surface equivalent dose ratio between rat and adult humans. The SMYZ-H group was administered 11.88 g/kg/d, which is 4 times the clinically equivalent dosage. The SMYZ-L group was administered 2.97 g/kg/d, which is the clinical equivalent dosage. The sham-operated group was used as the control group and administered an equal volume of distilled water. Each group received 1 ml/100 g/d distilled water intragastrically, donepezil dissolved in liquid, or SMYZD once a day for 8 weeks.

### 2.6. Animal Model of VCI

The surgical procedures were performed using a well-established technique [18], and the rats were fasted for 12 hours before the operation. The rats were placed in a supine position on the surgical table and were anaesthetized by intraperitoneal (i.p.) injection of a 1% solution of sodium pentobarbital (50 mg/kg). The skin of the neck was disinfected with iodine, and a median incision was made, separating the subcutaneous tissue, muscle, common carotid artery (CCA), and external carotid artery (ECA). The right CCA proximal and right ECA were clamped using an arterial clip. A 1 ml syringe was inserted into the right side of the CCA, and 0.3 ml of KMG suspension containing 0.21 ml of KMG primary liquid was slowly injected. The KMG suspension was configured with the stock solution and normal saline at a ratio of 7 : 3. The rats were released immediately after the injection. The CCA arterial clip on the right side allowed the microsphere embolus to pass through the right internal carotid artery with the blood flow (internal carotid artery, ICA) entering the intracranial blood vessels, and then, the right ECA arterial clip was released. The microsphere emboli were passed through the right internal carotid artery (ICA) with blood flow entering the intracranial blood vessels, and then, the right ECA arterial clip was released. Then, another rat was injected with 0.3 mL of ICA saline. The bleeding was stopped by applying pressure and a small amount of penicillin powder, after which the wound was sutured; the rat was returned to the cage. Intramuscular injection of 40,000 U penicillin was used after each operation as an anti-infection treatment for 3 days, while wound healing was observed.

### 2.7. Morris Water Maze

The Morris water maze (MWM) apparatus, 120 cm in diameter and 50 cm in depth, was produced by the Institute of Pharmacology at the Chinese Academy of Chinese Medicine. The Morris water maze test was carried out after 2 months of gavage to assess the learning abilities of the rats based on memory. In this apparatus, the water was approximately 30 cm deep and 1 cm above the platform surface, and the platform was located in the fourth quadrant of the maze. The water was maintained at a temperature of 25 ± 1°C. Some ink was added to the water to make it dark and opaque, preventing the rats from identifying the platform in the tank. The whole experiment was divided into two parts: the place navigation test and the spatial probe test. For the place navigation test, the rats were individually placed in the water against the wall of the tank at the marked entry point 1/2 radian from the second quadrant of the tank wall. The time required for each rat to find the platform was recorded and considered the escape latency until they left the platform, and the swimming distance was determined by an autotracking system. The animals were allowed to swim for 90 seconds to search for the platform during each trial and rest on the platform for 10 seconds if they found it. For the animals that failed to find the platform, the escape latency was recorded as 90 seconds. All of the rats were trained once a day for four consecutive days before the experiment. On the fifth day, the escape latency evaluation index of the learning memory ability of the rats was recorded in the morning. The next day, the spatial probe test was performed to assess the spatial memory ability without the platform in place. After an entry point was chosen, the rats were released into the water facing the tank wall, and the number of times the rats crossed the platform site within 90 seconds was recorded. The MWM test protocol followed Cao's method [19]. After the MWM test, the rats were fasted for 12 hours, and serum and brain tissue samples were taken. The brain was removed immediately after transcardial perfusion with saline. The brains were cut into specimens of sagittal forms randomly harvested from the left or right sides on ice. Six specimens in each group were fixed in 4% neutral paraformaldehyde solution and sliced after paraffin embedding, and these specimens were observed after hematoxylin-eosin (HE) staining and immunohistochemistry. The hippocampi of the other specimens were isolated and stored in a freezer at −80°C to detect the related molecules by dual luciferase reporter, Western blotting, and real-time fluorescence quantitative polymerase chain reaction (RT-PCR).

### 2.8. Brain Tissue Sampling

The rats were anaesthetized by intraperitoneal injection, and immediately after decapitation, their brains were quickly removed on ice. Each group included 4 rats, and the right hemibrain was fixed in 4% paraformaldehyde for sectioning and subsequent immunohistochemistry staining. One layer of tissue (approximately 1 mm^3^ each) from the right cerebral cortex of 2 rats in each group was fixed in 4% paraformaldehyde for transmission electron microscopic observation; the rest of the brain tissue was separated from the cerebral cortex and hippocampus, wrapped in tin foil paper and numbered, and stored in liquid nitrogen for flash freezing. Then, the tissue was stored in a freezer at −80°C to prepare for the extraction of brain microvascular pericyte-related proteins.

### 2.9. Transmission Electron Microscope Ultra-Pathology Observation Experiment

The brain tissues were fixed, dehydrated, soaked, and embedded to form an embedded mass. Then, ultrathin slicer sectioning at 70 nm was performed, and the sections were attached to copper mesh, stained with uranium acetate solution for 30 min, and stained with citric acid dye for 30 min. The images were observed and photographed by transmission electron microscopy.

### 2.10. Extraction of Brain Microvascular Pericell-Related Proteins

The method for extracting brain microvascular pericyte-related proteins was improved by referring to the method described in the literature [20] as follows: approximately 250 mg of cortex tissue was harvested from the right side of the brain and treated on ice. TEVP solution (320 nmol/L sucrose) was added at 100 mg/ml, and 1 mM PMSF was added. The tissue was homogenized at 800 × g and centrifuged at 4°C for 15 min. The precipitate was discarded, and the supernatant was collected. After a second centrifugation at 4°C for 15 min at 800 × g, the supernatant was discarded, the precipitate was retained, and TEVP solution was added to a final volume of 2 ml and mixed well. Then, the solution was placed on ice for 40 min, 1 mM PMSF was added, and the sample was placed in the freezer at −80°C for later use. The composition of the TEVP solution was 5 mmol/L Tris-HCL, 2.5 mmol/L NaF, 0.5 mmol/L Na3VO4, 0.5 mmol/L EDTA, and 0.5 mmol/L EGTA, added to 500 mL pure water. This solution was mixed and stored at room temperature.

### 2.11. Western Blotting

Six samples from each group were used to extract the tissue protein, and protein quantification was conducted by a BCA kit. Then, the protein concentrations were adjusted with 5x buffer solution. After mixing, the protein concentrations of the samples were adjusted to 4 mg/mL. According to the molecular weights of the target proteins, 12% and 8% separation gels were prepared; the concentration of the spacer gel was 5%; and the concentration of SDS stock solution and APS stock solution was 10%. For the electrophoretic buffer solution, 100 mL 10× Tris-glycine electrophoresis buffer was mixed well with ultrapure water to a final volume of 1 L and stored at room temperature. For the transfer buffer, 100 mL wet transfer buffer and 200 mL formaldehyde were combined, ultrapure water was added to 1 L, and the solution was mixed well and stored at room temperature. To prepare the TBST rinse solution, 100 mL 10x TBST was combined with 1 liter of ultrapure water, mixed well, and stored in refrigerator at 4°C. For electrophoresis, the spacer gel constant voltage was set to 90 V for approximately 20 minutes. The separation gel constant voltage was set to 160 V, and the electrophoresis was stopped based on the predyed marker protein. Then, the proteins were transferred onto the membranes, blocked with 3% BSA-TBST, and incubated with primary antibodies (FDGFR-*β* rabbit monoclonal antibody monoclonal antibody diluted to 1 : 1000 and NG2 rabbit polyclonal antibody diluted to 1 : 500) at 4°C overnight and the membranes were washed. The membranes were incubated with the internal reference GAPDH mouse monoclonal antibody and goat anti-mouse IgG (H & L) HRP antibodies. After incubation with the secondary antibodies, the membranes were washed again. After the ECL reaction, the film was exposed, developed, and fixed.

### 2.12. RT-PCR

Three samples from each group were selected, and total RNA was extracted with a TRIzol total RNA extraction kit. Primers were synthesized by Beijing Invitrogen Corporation. The upstream PDGFR primer was 5-GGCTGCTGGAAACACTGGA-3, and the downstream PDGFR primer was 5-AGAGGGCGTCGGATAAGC-3. The upstream NG2 mRNA primer was 5-AGCAGAATCTAGGACCAACCA-3, and the downstream NG2 mRNA primer was 5-GGTAAGGCTCAGTGGCAAAGT-3. The upstream GAPDH mRNA primer was 5-TTCCTACCCCCAATGTATCCG-3, and the downstream GAPDH mRNA primer was 5-CCACCCTGTTGCTGTAGCCATA-3. The extracted perivascular proteins were transferred to a 1.5 ml EP tube containing 1 mL TRIzol at room temperature and incubated for 5 minutes. Then, 0.2 ml of chloroform was added, shaken, mixed, and incubated at room temperature for 5 to 10 minutes. After centrifugation at 4°C for 15 minutes and 12,000 RPM, the supernatant water phase was transferred to new EP tube. The same volume of isopropanol was placed at 20°C for precooling. The solution was mixed and incubated on ice for 10 minutes. After centrifugation at 4°C for 15 min at 12,000 RPM, the supernatant was discarded, 75% DEPC ethanol was added at a ratio of 1 mL/mL TRIzol, and the mixture was oscillated to wash the precipitate. At 12,000 RPM, the sample was centrifuged at 4°C for 15 minutes and then the precipitate was washed once again. The ethanol liquid was discarded, the sample was incubated at room temperature for 5 min, and then, DEPC was added to treat the water and dissolve the precipitate. The RNA concentration and purity were determined using a NanoDrop® nd-2000, and the RNA was dissolved in DEPC-treated water prior to measurement. Denaturing agarose gel electrophoresis was performed at a voltage of 5-6 V/cm. cDNA reverse transcription was performed using the Prime Script™ RT reagent Kit with gDNA Eraser, and amplification was conducted with RealSuper Mixture (with Rox). The amplification procedure was as follows: 95°C for 10 min, 40 cycles of melting for 15 seconds at 95°C, and annealing and extension for 1 min at 60°C. The melting curve was analyzed at 60–95°C, after which the standard curve of the target genes and internal reference gene was generated. Finally, the data were quantified and analyzed using the 2 − ΔΔ*CT* method.

### 2.13. Statistical Analysis

The data are expressed as the mean ± standard error of the mean (SEM). All the statistical analyses were performed using the statistical package SPSS 17.0. One-way analysis of variance (ANOVA) with a least significant difference test or one-way ANOVA with post hoc Dunnett's T3 (multiple comparisons) was used to compare between-group values. *p* < 0.05 was considered statistically significant.

## 3. Results

### 3.1. SMYZD Ameliorates Spatial Learning and Memory Deficits in Rats with VCI

Rats with VCI were subjected to MWM navigation trials. On the first two days, there was no statistically significant difference between the groups in terms of escape latency (EL) (*p* > 0.05). Compared with that on the first day, the EL of all the rats on the fourth day of testing was significantly shortened (*p* < 0.05), indicating that all the rats had the ability to learn. According to repeated measurement variance analysis, compared with that in the control group, the EL in the model group was increased significantly (*p* < 0.05), suggesting that the spatial learning ability of the rats in the model group was significantly decreased. Compared with that in the model group, the ELs in the SMYZ-H group, SMYZ-L group, and donepezil hydrochloride group were decreased significantly (*p* < 0.05), suggesting that SMYZD could improve the spatial learning abilities of the rats with VCI.

**Figure 1:**
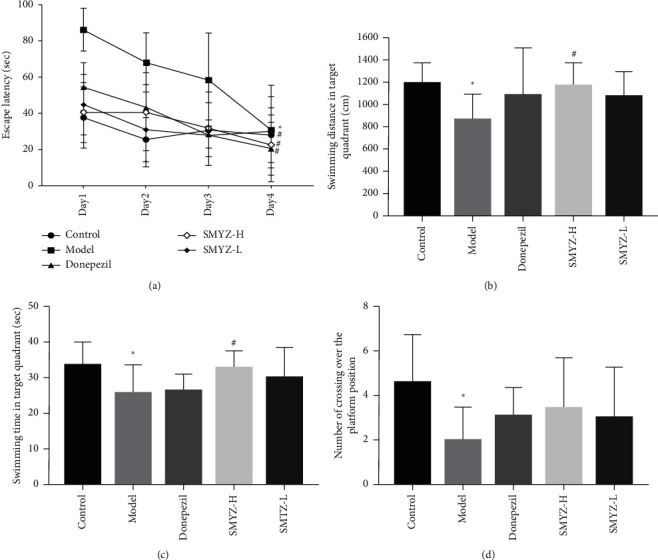
SMYZD ameliorated learning and memory deficits in rats with VCI. (a) All the groups displayed a spatial learning effect (*p* < 0.05). On the last day of training, the model group exhibited longer latencies to reach the platform than the control group (*p* < 0.05). Moreover, compared with the model group, the donepezil, SMYZ-H, and SMYZ-L groups exhibited shorter latencies to reach the platform on the fourth day (*p* < 0.05) of training. Compared with those in the control group, the values representing swim distance (b), time spent in the target quadrant, (c) and the number of crossings (d) were all significantly decreased in the model group (*p* < 0.05). Compared with those in the model group, the swim distance and time spent in the target quadrant in the SMYZ-H group were increased significantly (*p* < 0.05).  ^*∗*^*p* < 0.05 compared with the sham group; ^#^*p* < 0.05 compared with the model group. ^▲^*p* < 0.05 compared with the model group.

The MWM spatial probe trials showed that, compared with the control group, the model group exhibited shortened swimming distances and time spent in the target quadrant, and the frequency with which the rats crossed the platform was reduced significantly (*p* < 0.05). Compared with those of the model group, the swimming distance and time spent in the target quadrant of the SMYZ-H group were significantly prolonged (*p* < 0.05). Compared with that of the model group, the number of platform crossings increased in each group, and the swimming distance and time spent in the target quadrant of the SMYZ-L group were increased, but the difference was not statistically significant (*p* > 0.05). The results are shown in Figure 1.

### 3.2. SMYZD Improves Ultrastructural Changes in the Brain Microvasculature in Rats with VCI

**Figure 2:**
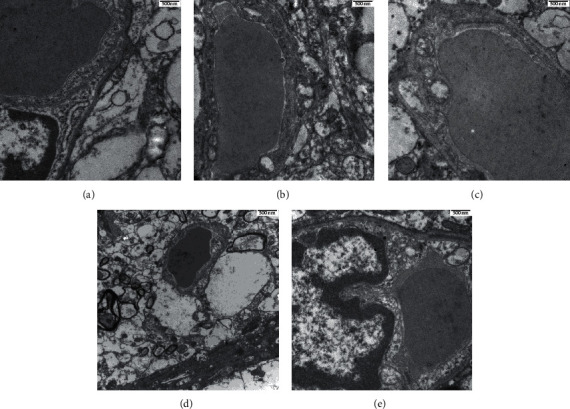
Effects of SMYZD on the ultrastructure of the brain microvasculature in the rats with VCI in each group. (a) Control. (b) Model. (c) Donepezil. (d) SMYZ-H. (e) SMYZ-L (bar = 500 nm).

Ultrastructural and ultrapathological changes in capillaries in the cerebral cortex were observed by transmission electron microscopy. The results showed that, in the control group, the morphology of the capillary endothelial cells was normal, the nucleus was clear, the vascular lumen was normal, and the thickness of the basement membrane was consistent. In the model group, the capillary endothelial cells were significantly swollen, the nuclear chromatin was homogenized, the vascular lumen was relatively narrow, fibrin was deposited outside of the capillary, and the basement membrane was thickened. In the donepezil group, SMYZ-H group, and SMYZ-L group, the endothelial cells were slightly swollen, the nuclei were pitted to different degrees, the chromatin margin was set (the nuclear chromatin margin set was obvious in the donepezil group), the vascular lumen was slightly narrowed, and the basement membrane thickness was different. The results are shown in Figure 2 (X20000).

### 3.3. SMYZD Changes the PDGFR-*β*, NG2, VEGF-A, and Ang1 mRNA Levels in the Brain Pericytes of Rats with VCI

**Figure 3:**
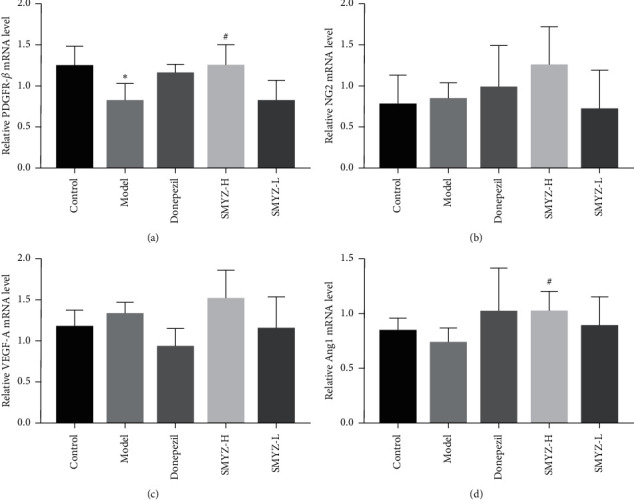
Effects of SMYZD on the mRNA levels of PDGFR-*β* (a), NG2 (b), VEGF-A (c), and Ang1 (d) in rats with VCI ( ^*∗*^*p* < 0.05 compared with the sham group; ^#^*p* < 0.05 compared with the model group).

RT-PCR was used to detect the mRNA levels of PDGFR-*β*, NG2, VEGF-A, and Ang1 in the BPCs of the rats with VCI. Compared with that in the model group, the mRNA levels of PDGFR-*β* and Ang1 in the SMYZ-H group were increased significantly (*p* < 0.05). Compared with the control group, the model group did not exhibit significant differences in the NG2, VEGF-A, and Ang1 mRNA levels (*p* > 0.05). Compared with those in the model group, the NG2 and VEGF-A mRNA levels in the SMYZ-H group showed an increasing trend, but the difference was not statistically significant (*p* > 0.05). The results are shown in Figure 3.

### 3.4. SMYZD Changes the Protein Expression of PDGFR-*β*, NG2, VEGF-A, and Ang1 in the Brain Pericytes of Rats with VCI

**Figure 4:**
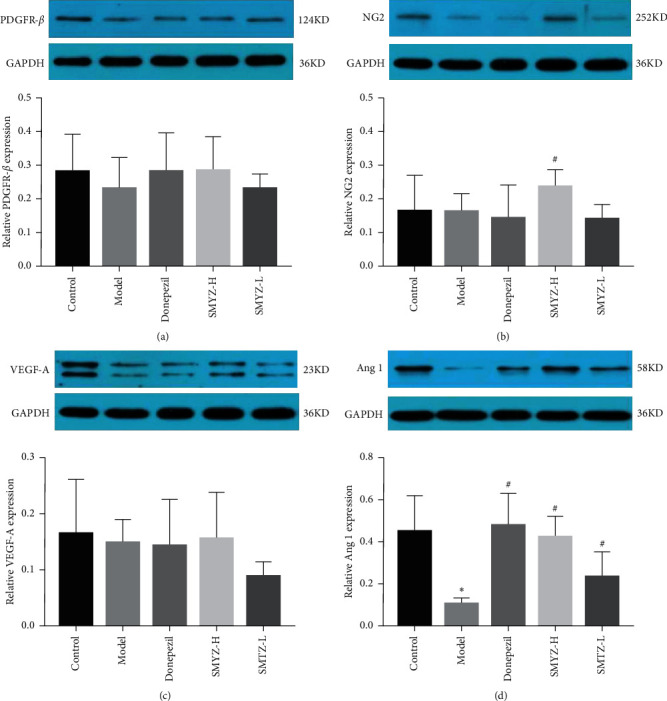
Effects of SMYZD on the expression of PDGFR-*β* (a), NG2 (b), VEGF-A (c), and Ang1 (d) in rats with VCI ( ^*∗*^*p* < 0.05 compared with the sham group; ^#^*p* < 0.05 compared with the model group).

The WB method was used to detect the protein expression of PDGFR-*β*, NG2, VEGF-A, and Ang1 in the BPCs of rats with VCI. The results showed that, compared with that in the control group, the protein expression of Ang1 in the model group was decreased significantly (*p* < 0.05) (Figure 4(d)), and the protein expression of PDGFR-*β*, NG2, and VEGF-A in the model group showed a decreasing trend, but the difference was not significant (*p* > 0.05) (Figures 4(a)–4(c)). The results are shown in Figure 4.

## 4. Discussion

The model established by the vascular embolization method can better simulate the infarction of deep and small parts of the brain and lacuna observed in clinical practice, and these phenomena are closely related to the occurrence of clinical VCI. The modeling method selected in this study was microsphere embolization, which includes the injection of sterile, standardized, mass-produced microspheres of uniform particle size (100–200 *µ*m in diameter) in a sodium alginate microsphere vascular embolic agent suspension to induce VCI. Since infarcts are not fixed, infarcts are not uniform in size and have characteristics of universality and unevenness. Most studies have focused on apparent strokes caused by blockages in major blood vessels; however, few studies have focused on small-vessel lesions, which often evolve into stroke and eventually lead to VCI [21]. Therefore, in this study, we observed the ultrastructure of small vessels in the cerebral cortex. The results show that there is obvious cerebral microvascular injury in rats with VCI, while, after SMYZD intervention, the ultrapathological structure of cerebral microvessels was improved.

Spatial learning and memory in rodents involves multiple brain regions and neural pathways, including the hippocampus, striatum, and basal forebrain [22]. The MWM has multiple advantages, including minimal training and ease of experiment operation, and the MWM is the most widely used tool for assessing and comparing rodent spatial learning and memory abilities [23, 24]. During the experiment, the spatial learning and memory abilities of the animals were assessed according to the escape latency (EL) period required to find the platform, the number of platform crossings, and the swimming distance and time spent in the target quadrant. The MWM results of this study showed that, compared with the control rats, the rats with VCI showed significant learning and memory dysfunction, as shown by the prolonged EL period, the reduced frequency with which the platform was crossed, and the increased distance traveled and time spent in the original platform quadrant. However, after 4 days of navigation training, the EL of the rats in each group was shortened, indicating that the rats exhibited improvements in their learning ability. After treatment with donepezil hydrochloride and SMYZD at high and low doses (SMYZ-H and SMYZ-L), the EL of the rats was significantly shortened compared with that of the model rats, indicating that SMYZD could improve the learning and memory abilities of rats with VCI. In addition, after treatment with high-dose SMYZD, the distance traveled and time spent in the original platform quadrant by the rats with VCI were significantly increased, indicating that SMYZD could improve the spatial cognition ability of rats with VCI.

The concept of the neurovascular unit (NVU) was formally established in 2001 by the Stroke Progression Review Group meeting of the National Institute of Neurological Disorders and Stroke, at which time the close relationship between the brain and its blood vessels was highlighted [25]. The protection of the NVU can reduce the damage to brain tissue after cerebrovascular disease. Neurovascular units, pericytes, and BBB-forming endothelium play important pathophysiological roles in ischemic and hemorrhagic cerebrovascular diseases [26].

Pericytes, a cell type located on capillaries, were first identified in the 19^th^ century by Rouget and are of crucial importance for regulating diverse microvascular functions, such as capillary blood flow, the blood-brain barrier, and angiogenesis [27]. Understanding pericyte-related vascular pathobiological events is pivotal not only to develop more tailored treatments of disease but also to establish pericytes as a therapeutic tool.

The interaction between pericytes and endothelial cells (ECs) is crucial for the integrity and maintenance of the basal membrane of the vascular wall. The contact between pericytes and ECs enables the pericytes to regulate blood flow in the vessels. Paracrine pericytes affect the proliferation and maturation of ECs, and they can promote the generation of new vascular buds when appropriate or inhibit the abnormal proangiogenic behavior of ECs when there is no need for vascular germination [28]. Pericytes mechanically regulate the integrity of vascular walls and act as signal-transmitting media to regulate EC behaviors [29]. Brain microvascular pericytes, or brain pericytes (BPCs), are important components of the blood-brain barrier (BBB), are located between ECs, astrocytes and neurons [30], and play an important role in maintaining cerebrovascular homeostasis to protect the central nervous system (CNS) from damage. BPCs not only contribute to the formation and maintenance of the BBB under physiological conditions but also contribute to the survival of ECs and the protection of the BBB after cerebrovascular injury under pathological conditions. In the cases of cerebral vascular injury and hypoxia, BPCs exerted a compensatory effect on protecting ECs and stabilizing the BBB [31]. The above studies showed that the stress response of BPCs to cerebral ischemic injury is dynamic and plays a positive role in NVU maintenance after injury. In addition, BPCs have extensive spatial distribution, diverse cell characteristics, and a close relationship with NVUs in brain microvessels, so the use of BPCs as a new therapeutic target for cognitive impairment after cerebrovascular damage is feasible.

PDGFR-*β* is a specific marker of CNS BPCs [32]. Compared with that in patients with normal cognition, the CSF level of PDGFR in patients with mild cognitive impairment was significantly increased, and the hippocampal PDGFR level in these patients was positively correlated with BBB permeability [33]. Therefore, PDGFR could be used as a reliable marker of CNS BPCs in in vitro animal and clinical studies. The loss of BPCs expressing PDGFR-T leads to an interruption of PDGF-B/PDGFR signals. Several studies using transgenic animal models harboring PDGFR-i-derived genes have demonstrated the functional effects of disruption of the PDGF-B/PDGFR-initiated signaling pathway [34, 35]. A recent study of PDGFR-*β* +/− mice, which show a reduction in brain BPC number of approximately 25%, found that reduced BPCs and early degeneration attenuated the CBF responses to neuronal stimulation, leading to neurovascular decoupling and reduced brain oxygen supply [34]. Studies of PDGFR-*β* signal transduction in PDGFR-.CITE.DATA-*β* +/− mice, which exhibit a reduction in brain BPCs of approximately 25%, have shown that BPCs in the cerebral cortex, hippocampus, and striatum exhibited early and progressive loss, leading to microvasculopathy and BBB destruction, which were manifested as decreased BPCs coverage and cell number, decreased capillary length, and increased perivascular fibrinogen and fibrin deposition [35].

Due to the diversity of the expression of PC markers, multiple markers (at least 2 markers) were used to identify PCs in vivo [36]. NG2, a proteoglycan that plays an important role in the proliferation, migration, and survival of BPCs, has become one of the most commonly used markers of BPCs in recent years [37].

In this study, PDGFR played a role in the proliferation, migration, and survival of BPCs and was induced by SMYZD, and its correlation with perivascular cells in the brain was discussed. The results showed that rats with VCI suffered significant brain microvascular injury, PDGFR-*β*-positive cell loss, and decreased PDGFR-*β* mRNA levels, suggesting that BPCs underwent denaturation or death after brain microvascular injury. After SMYZD treatment, the brain microvascular ultrapathological structure was improved, the number of PDGFR-*β*-positive cells increased, and the mRNA level of PDGFR-*β* mRNA increased significantly. The protein expression level of NG2, another protein marker of BPCs, increased significantly, and the mRNA level of NG2 showed an increasing trend, suggesting that SMYZD had a certain protective effect on BPCs in rats with VCI and might improve the damaged brain microvascular and brain microenvironment by protecting BPCs.

Vascular regeneration is crucial for the occurrence and development of cardiovascular and cerebrovascular diseases and tissue damage repair. Vascular regeneration is a complex biological process that includes the activation, chemotaxis, and proliferation of vascular constituent cells, the recruitment of microvascular peripheral cells (PCs), the coverage of ECs by PCs, and the maturation of new blood vessels [38]. PCs play an important role in the regulation of vascular regeneration [39]. After the occurrence of cerebrovascular injury, the expression of angiogenesis-related genes and various angiogenic factors, including vascular endothelial growth factor (VEGF), angiogenin (ANG), platelet-derived growth factor (PDGF), and transforming growth factor (TGF-*β*), is upregulated in human or rodent brain tissues [40]. VEGF is a key factor in vascular regeneration, and as a vasoactive substance, it plays a strong role in promoting vascular growth and increasing EC permeability [41]. VEGF can induce vascular proliferation in the injured brain, which is of great significance for improving brain repair after cerebrovascular injury. In addition, the VEGF/VEGFR2-mediated signaling pathway is involved in nerve regeneration and repair by triggering a variety of downstream signals [42, 43]. BPCs secrete VEGF in a paracrine manner, and there are many subtypes of VEGF. VEGF-A is the main component of the VEGF family involved in regulating vascular regeneration [44, 45]. In this study, the VEGF content in the serum and the VEGF protein and mRNA expression in BPCs of rats with VCI were detected. The results showed that SMYZD could increase the content of VEGF in the serum of rats with VCI, and by promoting the generation of VEGF, it can participate in the vascular regeneration of rats with VCI. However, SMYZD treatment did not significantly change the protein and mRNA expression of VEGF in the BPCs of rats with VCI, meaning that it had no obvious effect on the secretion of VEGF by BPCs. It is speculated that SMYZD may participate in the promotion of vascular regeneration by enhancing the secretion of VEGF by other CNS cells.

Ang1 derived from PCs participates in the process of vascular regeneration by activating the specific Tie2 receptor on ECs [46]. The Ang-Tie signal transduction system also participates in the communication between ECs and PCs. The combination of Ang1 and Tie2 stimulates the coverage of PCs and the deposition of basement membrane proteins, promotes the secretion of adhesion proteins, increases the interaction between ECSS, reduces the leakage of blood vessels and their permeability, and promotes the final maturity of new microvessels [47]. In this study, after investigating whether SMYZD can promote the stability and maturity of neovascularization by increasing the expression of Ang1 in the BPCs of rats with VCI, the protein and mRNA expression of Ang1 in the BPCs of rats with VCI was detected. The results showed that the protein expression of Ang1 in the BPCs of rats with VCI was significantly decreased, meaning that the decrease in Ang1 expression might cause vascular injury and neovascularization instability in rats with VCI. After SMYZD treatment, the relative protein and mRNA expression of Ang1 in the BPCs was significantly increased, suggesting that SMYZD can improve the maturation and stability of neovascularization in rats with VCI by promoting the expression of Ang1 in BPCs.

Analysis of the SMYZD water extract by HPLC has shown that its bioactive components include gastrodin, ginsenosidesRg1, ginsenosides Rb1, ferulic acid, and quercetin, all of which have effects in the treatment of cognitive disorders [13]. Studies have shown that gastrodin can inhibit the apoptosis of microvascular endothelial cells, reduce the infarct area, increase the formation of capillaries [48], and induce neuroprotection in mice with ischemic stroke [49]. In addition, early treatment with gastrodin can enhance the protein expression of VEGF in rats with motor learning disabilities to improve their cognitive function [50]. Panax ginseng has shown particular efficacy in the clinical treatment of certain diseases, such as dementia, diabetes, and respiratory infections [51]. Regarding the cognitive performance of subjects with probable AD, ginseng significantly improved their performance on both the ADAS-Cog test (which evaluates language, memory, attention praxis, and other cognitive abilities) and MMSE test (which evaluates language, attention, orientation, calculation, and basic motor skills) [52]. Other evidence shows that ginsenoside Rg1 decreases oxidative stress and reduces cognitive impairment in mice [53]. In addition, ginsenoside Rg1 has been proven to be effective against ischemia/reperfusion neuronal injury [54]. Ferulic acid has a protective effect on hypoxic-ischemic brain injury in rats [55]. Quercetin can reduce endothelial injury in the inflammatory vascular system [56] and attenuate vascular calcification by blocking oxidative stress [57]. Moreover, quercetin can also alleviate the cognitive impairment caused by ischemia-reperfusion and oxidative stress [58, 59].

In conclusion, our findings demonstrated that SMYZD may exert protective effects against learning and memory impairments in rats with VCI via the inhibition of pericyte degeneration in the brain. Our findings provide a solid foundation for future investigation of the role of pericytes in VD progression. As such, the traditional Chinese medicine SMYZD is a promising candidate for the development of novel VD treatments.

## Data Availability

The data are available upon request to the first author by email.
